# Using Cultivated Microbial Communities To Dissect Microbiome Assembly: Challenges, Limitations, and the Path Ahead

**DOI:** 10.1128/mSystems.00161-17

**Published:** 2018-03-27

**Authors:** Benjamin E. Wolfe

**Affiliations:** a Department of Biology, Tufts University, Medford, Massachusetts, USA

**Keywords:** cultivated, microbial communities, model system, microbiome assembly, metagenomics

## Abstract

As troves of microbiome sequencing data provide improved resolution of patterns of microbial diversity, new approaches are needed to understand what controls these patterns. Many microbial ecologists are using cultivated model microbial communities to address this challenge. These systems provide opportunities to identify drivers of microbiome assembly, but key challenges and limitations need to be carefully considered in their development, implementation, and interpretation. How well do model microbial communities mimic *in vitro* communities in terms of taxonomic diversity, trophic levels, intraspecific diversity, and the abiotic environment? What are the best ways to manipulate and measure inputs and outputs in model community experiments? In this perspective, I briefly address some of these challenges on the basis of our experience developing fermented food model communities. Future work integrating genetic and molecular approaches with cultivated model microbial communities will allow microbial ecology to develop a more mechanistic understanding of microbiome diversity.

## Abstract (precis)

As troves of microbiome sequencing data provide improved resolution of patterns of microbial diversity, new approaches are needed to understand what controls these patterns. Many microbial ecologists are using cultivated model microbial communities to address this challenge.

## PERSPECTIVE

In the mid-1900s, the field of plant ecology was at a turning point. After decades of developing methods and metrics to describe patterns of plant diversity, an understanding of the processes that shaped this diversity was still largely unknown. Most studies of plant communities were observational surveys across environmental gradients or across time and used statistical approaches to infer what processes might structure plant community composition (1). To develop a mechanistic understanding of plant community assembly, many plant ecologists in Europe, the United States, and Australia turned to simple, accessible, and widespread old-field plant communities. These abandoned agricultural fields were not “natural” plant communities, as they assembled on highly disturbed land and were composed of both native and nonnative species. But they were surprisingly reproducible and relatively low in diversity and could easily be recreated and manipulated in a controlled manner (2). Starting in the 1960s and continuing for the next several decades, experiments in old-field plant communities provided foundational knowledge on competition (3, 4), succession (5), the impacts of multitrophic interactions (6, 7), and relationships between biodiversity and ecosystem functioning (8).

Over the past several years, I have argued that microbial ecology faces a turning point similar to that which plant ecology faced in the mid-1900s (9–11). With the rise of high-throughput sequencing, patterns of microbial diversity in medical, agricultural, industrial, and natural ecosystems have emerged (12–14). But as others have discussed in recent reviews, a mechanistic understanding of the processes that shape the assembly of microbiomes is largely missing (15, 16). To better link patterns of microbiome diversity with specific processes, many labs are developing microbial equivalents of old-field plant communities. From tree holes in the United Kingdom to wheels of cheese throughout the world, we are culturing the members of widespread, accessible, and reproducible microbiomes to experimentally recreate and manipulate these communities in controlled environments (17). With these systems, individual species can be combined into experimental communities with specific compositions or conditions and community assembly can be monitored through time. These cultivated model microbial communities have the potential to link patterns of microbiome diversity with specific ecological and evolutionary processes, including dispersal, selection, diversification, and drift (16).

Cultivation of microbes from microbial communities is most certainly not a new endeavor, and many cultured microbial species have been used for decades to answer fundamental questions in microbiology and microbial ecology (18, 19). This new wave of cultivated model microbial communities has emerged from critical conceptual shifts in modern microbiology. There is a growing awareness that monocultures of model microbes studied in the lab cannot fully answer questions about the biology of multispecies microbial communities in nature. Another key problem that has spurred the resurgence of cultivated model microbial communities is that sequencing data alone cannot explain the patterns of microbiome diversity. You can analyze a metagenomic sequencing data set a multitude of ways, but the only way to fully understand what processes determine the composition of a microbiome is to carefully manipulate living organisms in a controlled environment.

My lab has been developing fermented food (cheese, sourdough, kombucha), cabbage leaf, and planarian worm cultivated model microbial communities in an effort to dissect patterns of microbiome diversity. Here, I present some of the most important lessons we have learned about the challenges and opportunities of these systems. Just as experiments using old fields could not fully represent all plant communities, cultivated model microbial communities have important limitations that need to be considered during the development, utilization, and interpretation of these systems.

One of the most important questions to ask during the development of cultivated model microbial communities is how well they represent microbial communities that reproducibly form outside the lab. In some cases, representing reality may not be the goal. Many highly synthetic model communities have been used to ask general questions about ecology and evolution that develop and test theory or techniques and make no attempt to mimic nature (17). Our work and this perspective focus on using model communities to explain patterns in naturally forming microbial communities, and in these cases, mimicking reality becomes more important. The balance between realism and reductionism will vary with the goals of the study and the constraints of each model system, but completeness of the communities, representativeness of trophic groups, intraspecific diversity, and abiotic realism are four key considerations that I discuss below.

Ideally, all of the taxa present *in situ* would be cultured and included in model microbial communities. Complete communities can be recreated for some low-diversity and easy-to-culture systems, but it is difficult to isolate all of the species in highly diverse microbial communities. In these more complex systems, representation in model communities can come at higher taxonomic levels, such at the genus or family level. For example, in complex host-associated microbial communities or high-diversity free-living communities, not all of the taxa detected by sequencing can be cultured, but often numerous representatives from across genera or families can be (20, 21). Including phylogenetically diverse representative taxa in model communities can help capture the high-level dynamics of community assembly, including patterns of succession (10), the structure of microbial interaction networks (22), and broad taxonomic responses to specific abiotic or biotic perturbations (23). It is important to note that using representative taxa assumes that key ecological traits are conserved at higher taxonomic levels, and this is very often not the case (24, 25). Communities that contain only a few strains to represent microbial genera or families may exclude species or strains with key ecological traits that play a role in community assembly. Moreover, using a synthetic community of 10 representative taxa to mimic a microbiome that normally contains 100 taxa may inherently miss many high-order interactions that could play key roles in community assembly (26).

Important insights into ecological and evolutionary drivers of microbial diversity can also be gleaned from model communities that include only a subset of all of the species detected *in situ*. These studies are often focused on explaining the ecology of a specific taxonomic group, such as co-occurring species within a genus, within the context of the larger complex community. For example, studies that have focused on *Streptomyces* communities in soil or *Vibrio* in marine environments have provided fundamental insights into the causes and consequences of competition within microbial species (27, 28). One major caveat against removing the background community is that ecological dynamics observed in a subset of the community in isolation will likely be very different from dynamics in the full community. For example, our own work on *Staphylococcus* in cheese rinds found that interactions among *Staphylococcus* species were highly dependent on the composition of the background microbial community (9).

Those studies that have tried to create “complete” communities often focus on just one major group of microbes in a particular system and ignore other microbial groups for the sake of simplicity. This is perhaps one the most significant limitations of many model cultivated microbial communities and something we should all work to rectify in the future. A majority of microbiome research has focused on bacterial communities. Bacteria are the dominant microbial members in the gut microbiome and some other well-studied environments, but many microbiomes have a high abundance of other microbial groups, including archaea, fungi, protists, and viruses. Because most of these groups have been studied by specialized labs with discipline-specific approaches to cultivation and manipulation, it is challenging for one lab to incorporate multiple microbial groups into most cultivated microbial communities. Yet observations from *in situ* communities suggest that there are interactions across these diverse microbial groups (29, 30). Incorporating these interactions into model systems is essential to capture the full range of dynamics within a community and to be able to best link *in situ* patterns with *in vitro* processes. For example, in our own work with cheese rind communities, bacteria are more diverse and sometimes more abundant than fungi (10), but in many cases, fungi determine the outcomes of community assembly in the system (9, 10). I appreciate the ease of reducing diversity down to specific groups, but the full utility and representativeness of cultivated model microbial communities will not be realized until we remove our taxonomic blinders.

Even in systems where all of the taxa can be easily cultured and the full range of microbial groups is included, representation of the genetic and phenotypic diversity within species is often lacking. Metagenomic studies across communities have often identified core microbiomes within systems, where the same dominant taxa consistently occur across many independent communities (31). Most model communities that have tried to recreate these core microbiomes have used microbial strains isolated from a single *in situ* microbial community. But we know from studies of microbial populations that there is substantial genomic and phenotypic diversity within microbial species isolated both within and across communities (32). How this strain level variation of core taxa impacts assembly dynamics has not been considered and should be incorporated in future experimental designs.

How well should the *in situ* environment be mimicked *in vitro* when using cultivated model microbial communities? For some experimental systems, precisely mimicking resource availability or abiotic conditions in a microbial community may not matter. For example, questions about how the rate or timing of dispersal affects community composition may not be dependent on nutrient availability (see reference 33 for exceptions). But studies on the impacts of abiotic and biotic selection on community assembly strongly depend on the environment. For simplicity, many experiments with cultivated model microbial communities have been performed under highly synthetic environmental conditions, such as in commercially available rich media or on agar plates. These simple and robust media have been used for decades in microbiology and are appealing because many microbes can easily grow on them. But these conditions rarely mimic the resource availability and biophysical space of natural microbial communities. To truly understand if a particular process identified *in vitro* is really relevant to *in situ* communities, we need to do a better job mimicking environments found *in situ*. One way to do this is to work with communities that grow in easy-to-reproduce and fairly defined substrates, which is what my group has been doing with fermented food microbial communities and others have done with nectar and aquatic communities (34, 35). Another approach is to develop synthetic media that mimic the resources available *in situ*. This is difficult work, as it requires complete chemical characterization of microbial ecosystems, but has been productive in mimicking environments of the human microbiome (e.g., see reference 36).

Researchers using cultivated model microbial communities should also carefully consider the best ways to measure and manipulate inputs of experimental communities. The standard approach for preparing microbial cells is to grow liquid cultures overnight and then harvest late-log- or stationary-phase cells. This lab approach may not be relevant to most naturally forming microbial communities, where cells are often in dormant states or grow at much lower rates (37). Recent work has also demonstrated that the method of preparing microbial cultures, such as overnight incubation in liquid versus scraping colonies from agar plates, can substantially impact the physiology of cultures in experiments (38). Standardizing the input cell concentrations can also be very difficult, as the same optical density of cells does not necessarily correspond to the same number of colony-forming units across different species or different types of microbes (e.g., bacteria, yeasts, and filamentous fungi). The best solutions to these challenges will likely depend on the systems being studied, but the use of media that mimic natural nutrient availabilities when preparing inocula and using frozen glycerol stocks that are dormant may be two approaches to these challenges.

Regardless of the specific question or system, another key challenge is considering the best way to measure outputs of experiments, including microbial community composition. To best link *in vitro* experimental community data with *in situ* metagenomic data, amplicon or shotgun sequence-based approaches can be used to quantify the composition of cultivated model microbial communities. But the power of culture-based communities is that we can detect and measure viable cells, which may provide a more meaningful metric of community composition than quantifying the total DNA extracted from cells. Culture-based approaches for quantifying microbial community composition are easiest when different taxa can be distinguished by using colony morphology or selective media. Even in systems where all of the taxa can be cultured, it is important to consider whether culture-based approaches will miss low-abundance species and how interactions between species on agar plates might impact the accuracy of quantifying all of the species present.

How well do processes and mechanisms identified in cultivated model microbial communities translate to naturally forming microbial communities? I would argue that we still have a long way to go. Keep in mind that many of these systems are highly synthetic and are not intended to mimic naturally forming communities. But for those systems that are intended to resemble some form of a natural microbiome, we are still discovering just how to best use these tools. Many systems have been used to identify potential processes or mechanisms in the lab, but it is rare to see confirmation that these processes or mechanisms are happening in natural systems. There are two general approaches to address this problem: (i) recreate highly controlled lab experiments in more complex natural microbiomes to demonstrate ecological relevance and (ii) link observations from the lab (e.g., species interactions) with microbiome sequencing data from the field (e.g., co-occurrence data). A great model of the first approach is the nectar microbial community of the flowering plant *Mimulus aurantiacus* developed by Tadashi Fukami at Stanford University. This system contains a relatively low number of yeasts and bacteria and has been successfully used in the lab to identify potential community assembly processes and in the field to confirm the ecological relevance of these processes (34, 39–41). A handful of studies have also linked experiments from cultivated microbial communities to *in situ* patterns of diversity in metagenomic sequencing data (9, 42, 43), but these studies are relatively rare.

Old-field plant communities did not solve all of the grand challenges of plant ecology and had many limitations. As I have just briefly described, cultivated model microbial communities also have caveats and limitations (too many to cover in this limited space) that will prevent them from solving all of the unresolved questions about microbial community assembly. Despite this, I believe the use of cultivated model microbial communities has a bright future. One productive path forward is a cross-biome approach, where multiple microbial systems are used to address the same questions or characterize the same phenomenon. Most labs have focused on one model system for their questions of choice. But the relative importance of an ecological or evolutionary process may be unique to a particular microbial community because of the specific taxonomic composition or environmental constraints of that community. The most powerful advances will come from identifying truly generalizable microbiome assembly phenomena that occur in many different types of microbiomes. This could come from using the same cultivated model microbial communities across different hosts/environments or from the use of different microbial communities that naturally vary in taxonomic composition or complexity. To allow for labs to easily test their findings in already established model systems, we should prioritize efforts to create easy-to-access and standardized libraries of cultivated model microbial communities.

I also strongly believe that the integration of cultivated model microbial communities with emerging genetic and molecular methods will provide a much needed mechanistic understanding of microbiome assembly. Many cultivated model microbial communities have been used primarily to quantify the relative impacts of ecological and evolutionary processes on community assembly, and the genes and molecules that regulate these processes are often not identified. By applying tools such as Barseq (44) and imaging mass spectrometry (45) to determine the genetic and molecular mechanisms that control assembly processes within cultivated model microbial communities, we will be able identify mechanisms that can be used to manipulate and manage microbiomes.
